# Biochemical analysis of human eIF4E-DCP2 interaction: Implications for the relationship between translation initiation and decapping

**DOI:** 10.1371/journal.pone.0322271

**Published:** 2025-08-01

**Authors:** Zachary F. Mandell, Jeff Coller

**Affiliations:** 1 RNA Innovation Center, Institute for NanoBioTechnology, Johns Hopkins University, Baltimore, Maryland, United States of America; 2 Department of Molecular Biology and Genetics, Johns Hopkins University, Baltimore, Maryland, United States of America; Korea University, KOREA, REPUBLIC OF

## Abstract

All eukaryotic mRNAs bear a 7-methylguanosine cap on their 5’ end. The 5’ cap enables mRNA translation by binding directly to eIF4E; which further recruits other factors and the 40S ribosome. Additionally, the 5’ cap maintains transcript stability; removal of the cap by the enzyme Dcp2 is necessary to degrade the mRNA. An *a priori* conclusion, therefore, has been that cap binding by eIF4E and DCP2 are antithetical to each other as both need access to the same substrate, i.e., the 5’ cap. In this study, we purified native full-length human eIF4E and Dcp2 and utilize biophysical and biochemical approaches to examine the in vitro interplay between Dcp2 and eIF4E. We confirm that Dcp2 is sufficient to remove the 5’ cap. Moreover, we demonstrate that Dcp2 binds RNA with nanomolar affinity. We discovered that, unexpectedly, eIF4E does not interfere with Dcp2’s decapping function, contradicting previous mechanistic models. Moreover, eIF4E binding appears to increase the affinity of Dcp2 for RNA. Although limited to in vitro conditions, our findings warrant a reevaluation of the proposed relationship between these mRNA cap-binding proteins.

## Introduction

A distinguishing feature of eukaryotic mRNA is the 7-methylguanosine (m7G) cap added co-transcriptionally to the 5’ end [1]. The 5’ m7GpppN cap serves two critical functions; it promotes ribosome association by binding the translational initiation factor eIF4E [2], and it protects the mRNA from degradation by blocking the activity of the 5’-to-3’ exoribonuclease, Xrn1 which acts preferentially on a 5’ monophosphate [3–5].

The control of mRNA stability is well recognized to be intimately connected with mRNA translation [4–10]; mRNAs that are decoded efficiently are more stable, while mRNAs that decode inefficiently are less stable. The first step in eukaryotic mRNA decay is removal of the 3’ polyadenosine tail by the CCR4/NOT complex [5]. This in turn allows for decapping of the 5’ cap by the DCP2 decapping enzyme and then digestion of the mRNA’s body 5’ to 3’ by XRN1 [5]. Much work has investigated the nature of how mRNA translation impacts mRNA decay rate [5]. In budding yeast, a major determinate of deadenylation and decapping rate is codon optimality which disproportionally alters elongation rate, allowing probing and binding of the ribosome by the CCR4/NOT complex [5]. In human cells, CCR4/NOT appears to also probe and bind the ribosome; codon specificity is also a determinant [5,11]. Following these events, a decapping complex must assemble on or in proximity to the 5’ cap structure, thereby allowing the cap to be enzymatically cleaved by DCP2. Importantly, since the process of mRNA occurs co-translationally [5], the presence of translational initiation factors on the 5’ end has theoretically been seen as a barrier to the decapping reaction [10]. Thus, it has been proposed that DCP2 binding and catalytic activity are mutually exclusive with eIF4E binding, although this has not been rigorously established [2,12]. A previous study suggested that an enzymatic mRNA decapping activity in yeast is inhibited in vitro by the addition of eIF4E [10], although this work predates the discovery of Dcp2 and thus requires reexamination.

Herein, we purified full-length human Dcp2 and eIF4E. We confirmed as previously reported that Dcp2 operates specifically on m7G capped RNA, with a turnover rate 5-fold greater than that of the yeast Dcp2:Dcp1 heterodimer [13]. This activity may potentially be explained by a tight binding affinity between human Dcp2 and RNA, as we find that Dcp2 exhibits an affinity of ~157 nM for m7G-capped RNA, which is approximately the same as the affinity of eIF4E for m7G-capped RNA [14,15]. Intriguingly, we find that Dcp2 binds ~1.6-fold more tightly to its enzymatic product, 5’ monophosphate RNA. Moreover, we find that eIF4E does not inhibit Dcp2 binding in our hands in vitro. In fact, eIF4E occupancy increases the affinity of Dcp2 for the RNA by ~2-fold. Lastly, we find that pre-incubation of the RNA with eIF4E did not significantly impact the rate of decapping by Dcp2. Together, our data provide a few new insights into the relationship between eIF4E and the human mRNA decapping enzyme that should be considered as we further elucidate the interplay between translation and mRNA decay.

## Results

### Human Dcp2 specifically removes the RNA 5’ m7G cap in the absence of decapping coactivators

Much is known about the activity of the eukaryotic decapping enzyme Dcp2 and its associated coactivators [16,17]. Dcp2 consists of an N-terminal regulatory domain (RD), a catalytic domain (CD), and a C-terminal intrinsically disordered region (IDR) [17]. The length of the RD and the IDR are highly variable across species and the various functions of these features are not entirely understood [17]. The CD is composed of a NUDIX hydrolase, which is a ubiquitous family of nucleotide hydrolases that typically utilize a metal cation to catalyze the hydrolysis of nucleosides [17]. In yeast, the C-terminal IDR auto-inhibits the enzyme [13] and binds additional mRNA decay factors [13,18–20]. In yeast, Dcp2 requires decapping coactivators, such as Dcp1, to achieve decapping [13,12,21–24]. Importantly, Dcp2 functions distinctly between eukaryotes. First, the C-terminal IDR of human Dcp2 is approximately 400 amino acids shorter than that of the yeast Dcp2 C-terminal IDR [12]. Second, Dcp2 does not form a heterodimer with Dcp1 in metazoa, rather these two factors interact indirectly via the scaffold protein Edc4 [25–27]. Third, biochemical studies of human Dcp2 have shown it does not require coactivators to achieve an appreciable level of decapping *in vitro* [14,28–30].

**Fig 1:**
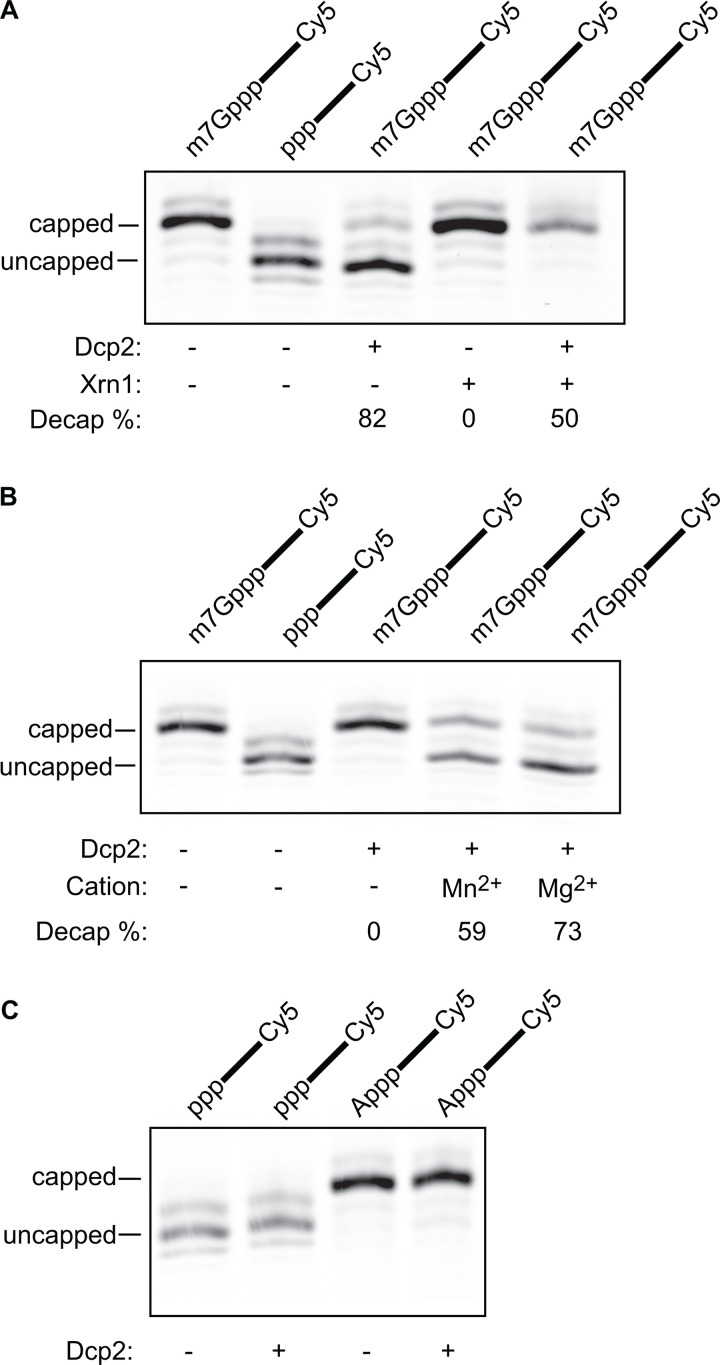
Human Dcp2 de-caps m7G-capped RNA. A. Decapping and decay reactions using RNA oligos containing the 5’ moieties that are indicated above each lane. Experiments were performed in the absence (-), or presence of Dcp2, and/or yeast Xrn1 as specified under each lane. The decapping % for the data shown here is shown underneath each lane. The capped and uncapped RNA species are indicated by the horizontal bars to the left of the gel slice. B. Decapping reactions using RNA oligos containing the 5’ moiety indicated above each lane. Experiments were performed in the absence or presence of Dcp2, Mg^2+^, and/or Mn^2+^, as indicated under each lane. The decapping % for the data shown here is shown underneath each lane. The capped and uncapped RNA species are indicated by the horizontal bars to the left of the gel slice. C. Decapping reactions using RNA oligo containing the 5’ moiety indicated above each lane. Experiments were performed in the absence or presence of of Dcp2 as indicated under each lane. The capped and uncapped RNA species are indicated by the horizontal bars to the left of the gel slice.

In order to understand the in vitro characteristics of the human decapping enzyme, we purified full-length Dcp2 to >90% purity (S1A Fig). We first tested enzyme activity by measuring decapping of a 43-mer RNA oligo that was modified at the 5’ end with m7GpppAm and labelled at the 3’ end with a Cy5 tag (S1B Fig). Purified Dcp2 was incubated for 30 minutes at 37°C with the synthetic RNA substrate. The reaction conditions were similar to those reported previously [28,29,31–33]. Resolution of the RNA substrate by PAGE revealed that incubation with Dcp2 changed the synthetic RNAs mobility, resulting in the appearance of a faster migrating species. This new species was comparable in size to a 43-mer RNA oligo that was identical in sequence, yet 5’ triphosphate (Fig 1A). Removal of m7GDP from the capped RNA was verified by incubation of the RNA with both Dcp2 and Xrn1. Xrn1 is a 5’ to 3’ exoribonuclease that acts specifically on 5’ monophosphate RNA [3]. Incubating the synthetic RNA with both Dcp2 and Xrn1 resulted in the disappearance of the faster migrating RNA species but not the slower species. These data suggest that the faster migrating species corresponded to RNA oligo that had been decapped by Dcp2 (Fig 1A). To further verify the activity of purified Dcp2, we incubated the capped RNA substrate in the absence of divalent cations, where we confirmed that Dcp2 requires the presence of divalent cations in the reaction buffer to catalyze decapping (Fig 1B). Intriguingly, we also noted that Mg^2+^ coordinated the reaction more efficiently than Mn^2+^ (Fig 1B, 73% vs. 59% decapped), which is inconsistent with previous reports using purified human Dcp2 [31]. Lastly, we confirmed that Dcp2-mediated catalysis is specific for the m7G cap. More specifically, we found that incubation of Dcp2 with RNA bearing either an ApppAm cap or 5’ triphosphate did not result in any alteration to the RNA (Fig 1C). Together, these results are consistent with previous observations that human Dcp2 decaps RNA in the absence of known decapping coactivators in vitro [28,29,31–33].

### Human Dcp2 displays affinity for RNA

**Fig 2:**
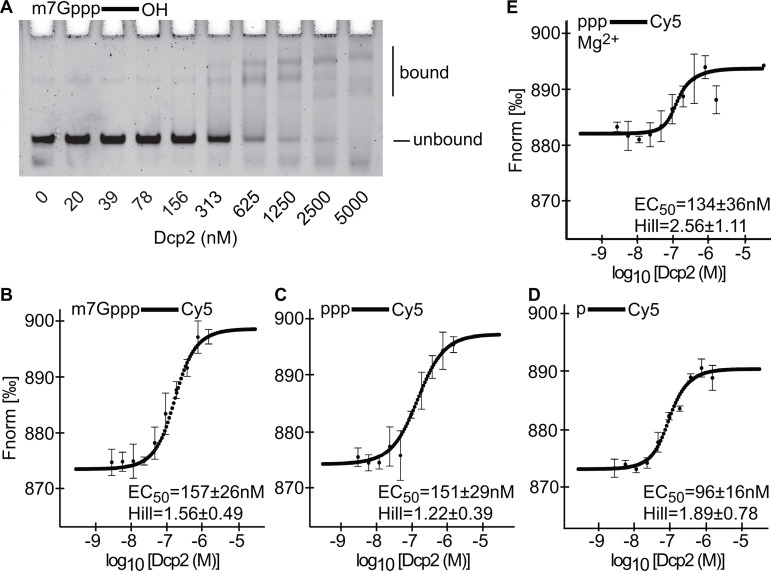
Dcp2 binds to RNA. A. Gel-shift assay. As indicated above the gel slice, m7G-capped 3’ unlabeled RNA oligos were incubated with the concentration of Dcp2 indicated under each lane. The bound and unbound fractions are specified by the bars to the right of the gel slice. B. MST assay using 3’ Cy5 labeled m7G-capped RNA. The Fnorm is specified on the y-axis, while the concentration of Dcp2 specified by the x-axis. Each circular point corresponds to the median Fnorm at that concentration of Dcp2, with the bars representing the s.d. for each data point, n = 3 biological replicates. The median EC_50 _± s.d. of the three replicates is indicated in the lower-right corner of the panel. The perforated line corresponds to the Hill model that was constructed based on the median of each datapoint. C. Same as panel A, except with 3’ Cy5 labeled 5’ triphosphate RNA. D. Same as panel A, except with 3’ Cy5 labeled 5’ monophosphate RNA. E. Same as panel C, except the binding buffer included Mg^2+^.

Human Dcp2 has been reported to bind to both capped and 5’ triphosphate RNA [25,14]. To determine the affinity of Dcp2 for RNA, we first conducted a gel-shift assay using m7G-capped, 3’ unlabeled RNA in the presence of varying concentrations of Dcp2 (Fig 2A). To ensure that Dcp2 did not de-cap the RNA during the reaction, we omitted a source of Mg^2+^ (see Fig 1B). We found that incubation of the RNA with Dcp2 led to multiple distinct bound species, indicating that Dcp2 binds to RNA in a multivalent stoichiometry. Intriguingly, at higher concentrations of Dcp2, we observed a variety of additional bands that were both more mobile and less mobile than the band corresponding to Dcp2 bound to RNA at a 1:1 ratio. While the exact cause of this pattern is unclear, one possible explanation is that at higher concentrations of Dcp2, this factor forms heterogenous phase-separated condensates with the RNA. Dcp2 has long been known to be a component of p-bodies, which are liquid droplet structures [20]. To measure the affinity of these interactions, we utilized MicroScale Thermophoresis (MST) [34]. While the K_D_ fit is appropriate to model binding interactions that follow a 1:1 stoichiometry, the Hill fit is better suited to model multivalent interactions [35]. Considering the multivalency between Dcp2 and the RNA (see Fig 2A), we chose to model our MST data using the Hill fit. The Hill fit yields two binding parameters, the EC_50,_ which is a measure of binding affinity, and the Hill coefficient [35]. Hill coefficients greater than 1 indicate a positive cooperativity [34], or a promiscuity of binding [36]. Quantitation of binding affinity is contingent on experimental conditions [37]. Thus we refer to all EC_50_ values in this report as the observed EC_50_, or the EC_50,obs_. Using this method, we found that the EC_50,obs_ between Dcp2 and m7G-capped RNA is 157 ± 26 nM (Fig 2B). In addition, the Hill coefficient of this interaction was found to be 1.56 ± 0.49, indicating that Dcp2 may bind to RNA nonspecifically, as others have suggested [38].

To determine the extent to which the m7G cap drives the affinity of Dcp2 for RNA, we repeated the MST experiment using oligos that were 5’ triphosphate. Here, we observe that Dcp2 exhibited a EC_50,obs_ of 151 ± 29 nM for this RNA, with a Hill coefficient of 1.22 ± 0.39 (Fig 2C). Next, we repeated this assay using RNA oligos that were 5’ monophosphate (Fig 2D). In this case, we determined that Dcp2 exhibited a EC_50,obs_ of 96 ± 16 nM, with a Hill coefficient of 1.89 ± 0.78. Thus, the affinity of Dcp2 for the reaction product (i.e., a 5’ monophosphate) is ~ 1.6-fold stronger than the affinity of Dcp2 for the reaction substrate. To ensure that the lack of Mg^2+^ in the binding mixture did not majorly influence our results, we incubated Dcp2 with 5’ triphosphate RNA in our decapping buffer and repeated the MST assay, where we found that Mg^2+^ does not majorly influence the EC_50,obs_ of Dcp2 for this RNA (Figs 2C,E).

**Fig 3:**
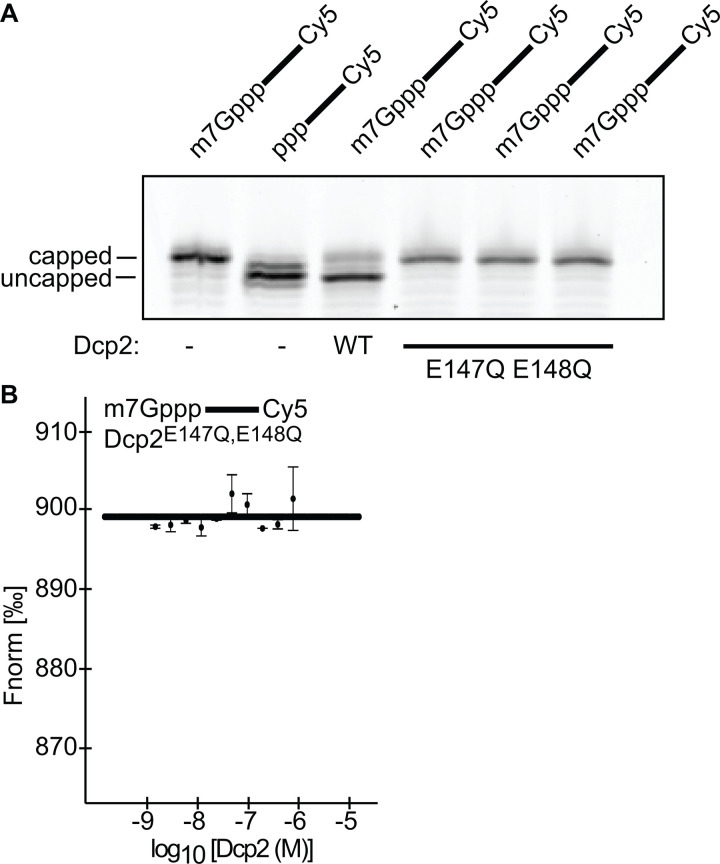
The NUDIX domain of Dcp2 participates in RNA binding. A. Decapping reactions using RNA oligos containing the 5’ moieties that are indicated above each lane. Experiments were performed in the absence (-), or presence of wild-type Dcp2, or mutant Dcp2, as specified under each lane. B. MST assay, with 3’ Cy5 labeled m7G-capped RNA. The Fnorm is specified on the y-axis, while the concentration of mutant Dcp2 specified by the x-axis. Each circular point corresponds to the median Fnorm at that concentration of Dcp2, with the bars representing the s.d. for each data point, n = 2 biological replicates. The perforated line corresponds to the non-binder model that was constructed based on the median of each datapoint.

Multiple reports have found that mutating key residues (E147, E148) within the catalytic center of the human Dcp2 NUDIX domain abrogates catalytic decapping (29,34). It has been suggested that the NUDIX domain promotes RNA binding [38]. To test this, we purified human Dcp2^E147Q,E148Q^ and confirmed that this enzyme cannot de-cap RNA *in vitro* (S1A Fig, Fig 3A). We next repeated our MST assay, applying this nuclease deficient Dcp2 to m7G-capped RNA, where we observed no binding (Fig 3B).

### Dcp2 binds to and de-caps m7G-capped RNA oligos that are bound to eIF4E

In the cytosol, the 5’ cap is associated with the translational initiation factor, eIF4E [2]. The contribution of bound eIF4E to mRNA decapping is unclear, although previous reports have found that eIF4E inhibits yeast decapping activity *in vitro* [10]. To test the effect of eIF4E on Dcp2-mediated decapping, we purified full-length human eIF4E to >90% purity (S2A Fig). Using a gel-shift assay, we confirmed that purified eIF4E binds specifically to m7G-capped RNA and does not interact with RNA lacking a cap structure, i.e., a 5’ triphosphate RNA, or with A-capped RNA (S2B Fig).

**Fig 4:**
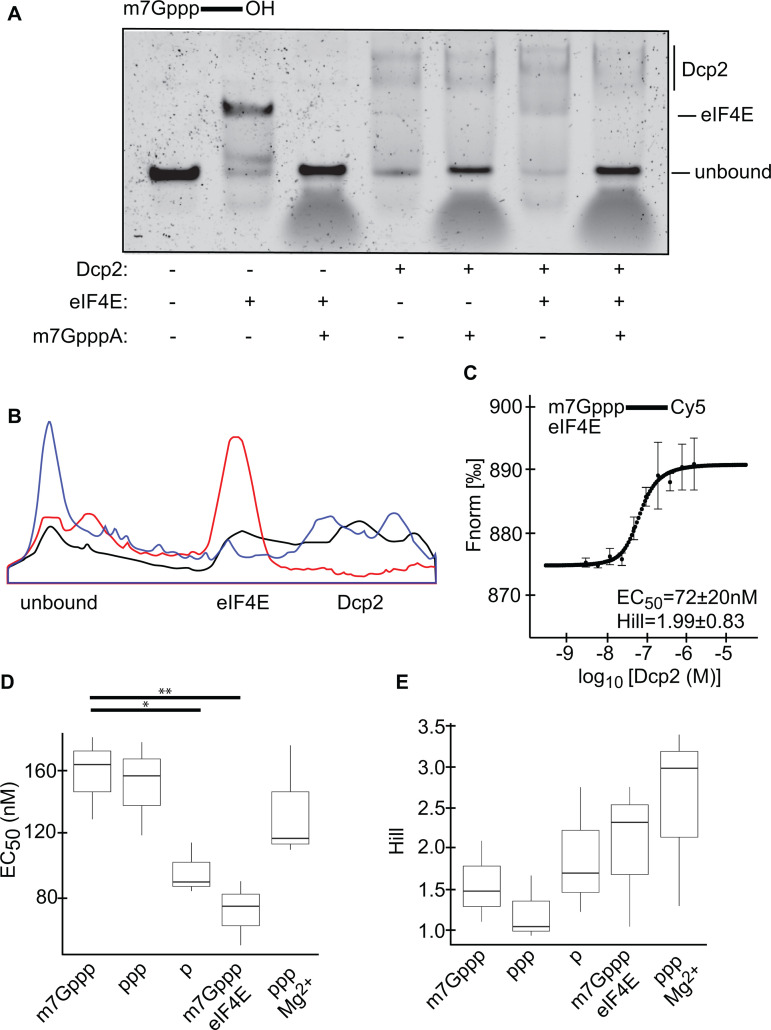
Dcp2 binds to RNA that is pre-bound to eIF4E. A. Gel-shift assays using m7G-capped 3’ unlabeled RNA oligos, as indicated above the gel slice. Experiments were performed in the absence or presence of Dcp2, eIF4E, and m7GpppA cap analog, as specified under each lane. The unbound fraction, the fraction bound singly to eIF4E, and the fractions bound to Dcp2 are specified by the bars to the right of the gel slice. B. Signal intensity was plotted across the + eIF4E lane (red), + Dcp2 lane (blue), and +eIF4E + Dcp2 lane (black) shown in panel A. C. MST assay, wherein m7G capped, 3’ Cy5 labeled RNA oligo and eIF4E, as indicated by the label in the upper-left corner, were incubated with a 2-fold serial dilution of Dcp2. The Fnorm is specified on the y-axis, while the concentration of Dcp2 specified by the x-axis. Each circular point corresponds to the median Fnorm at that concentration of Dcp2, with the bars representing the s.d. for each data point, n = 3 biological replicates. The median EC_50 _± s.d. of the three replicates is indicated in the lower-right corner of the panel. The perforated line corresponds to the Hill model that was constructed based on the median of each datapoint. D. Box plot of the EC_50 _± s.d. of Dcp2 for each of the substrates. Box, first to last quartiles; whiskers, 1.5 × interquartile range; centre line, median; points, outliers. Unpaired Wilcoxon rank sum tests were conducted, with results by the stars above the two horizontal black lines. E. Same as panel D, except showing the Hill coefficient± s.d. of Dcp2 for each of the substrates.

The effect of prebound eIF4E on Dcp2 RNA binding (see Fig 2) was then tested using a gel-shift assay. First, we incubated m7G-capped, 3’ unlabeled RNA with eIF4E, Dcp2, or no factor. We then resolved the bound and the unbound fractions using nondenaturing PAGE. Similar to the affinity experiments in Fig 2, we conducted these experiments in the absence of divalent cation to prevent catalytic decapping (see Fig 1B). In these conditions, we observed a distinct eIF4E shift and multiple Dcp2 shifts (Fig 4A). We repeated these two experiments in the presence of excess m7GpppA cap analog, where we confirmed that cap analog completely inhibits the binding of eIF4E to RNA, and mildly inhibits the binding of Dcp2 to RNA (Fig 4A). Next, to test the effect of prebound eIF4E on Dcp2 binding, we pre-incubated the oligo with eIF4E, after which we added Dcp2 to the same concentration as eIF4E. Here, we observed gel-shifts corresponding to eIF4E bound singly to the oligo, as well as Dcp2 bound to the oligo (Fig 4A). Intriguingly, we noted that the amount of eIF4E bound singly to the oligo was decreased by ~4.5-fold compared to the + eIF4E only condition. In addition, we noted that pre-bound eIF4E modulated the Dcp2 shift pattern. To better visualize these effects, we quantified the signal intensity across the + eIF4E, + Dcp2, and +eIF4E + Dcp2 lanes (Fig 4B). We then repeated the + eIF4E + Dcp2 binding condition in the presence of excess m7GpppA, where we again confirmed that cap analog inhibits eIF4E (Fig 4A).

To more quantitatively assay the effect of bound eIF4E on the affinity of Dcp2 for RNA, we pre-incubated m7G-capped, 3’ Cy5 labeled RNA with eIF4E, after which we incubated this mixture with a serial dilution of Dcp2. The affinity of Dcp2 for the RNA was then measured using MST, where we found the EC_50,obs_ between Dcp2 and the m7G-capped RNA was 72 ± 20 nM in the presence of eIF4E, with a Hill coefficient of 1.99 ± 0.83 (Fig 4C). Thus, eIF4E increases the affinity of Dcp2 for the RNA oligo by ~2-fold (Fig 3).

**Fig 5:**
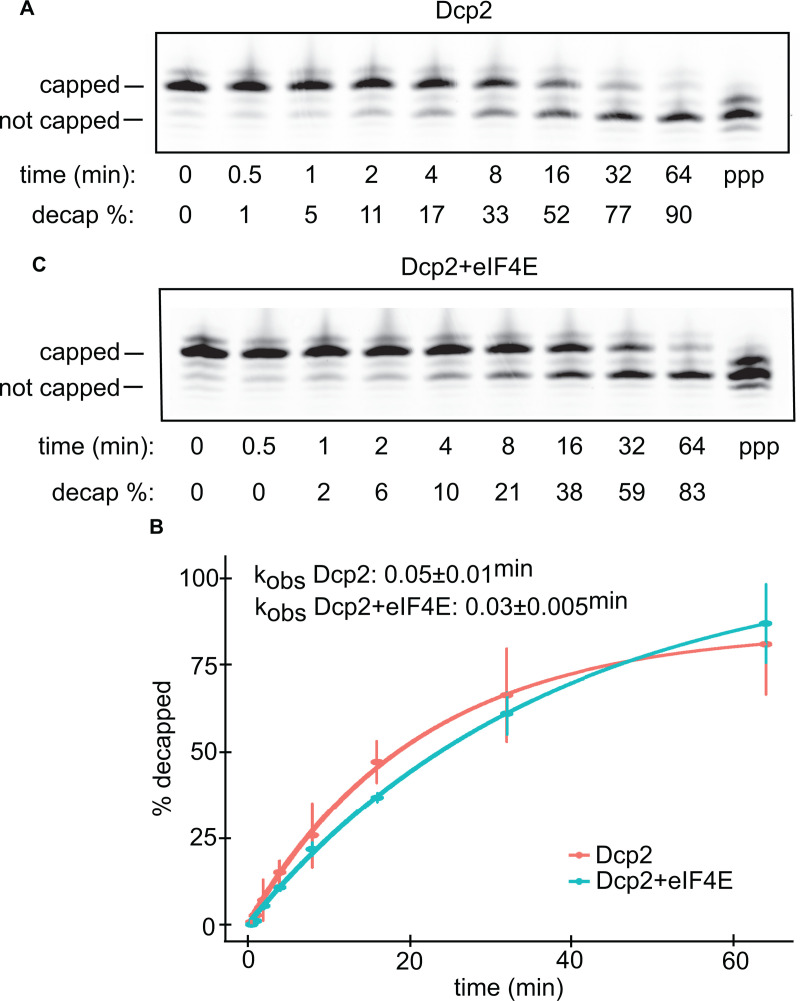
Dcp2 de-caps RNA oligos that are bound to eIF4E. A. Decapping time-course reactions using m7G-capped RNA oligos. RNA oligo was incubated with Dcp2 for the amount of time specified under each lane. The decapping % for the data is shown underneath each lane. The capped and not-capped RNA species are indicated by the horizontal bars to the left of the gel slice. The right-most lane contains 5’ triphosphate RNA oligo. B. Point-and-line plot of the decapping time-course data. Each circular point corresponds to the median % decapped after that amount of time with Dcp2, with the bars representing the s.d. for each data point, n = 3 biological replicates. The red data corresponds to the data collected using Dcp2, while the blue data corresponds to the data collected using both eIF4E and Dcp2. The median k_obs _± s.d. of the three replicates is indicated in the top left corner of the plot. C. Same as panel A, except showing the RNA oligo incubated with eIF4E prior to Dcp2.

Next, we tested the influence of prebound eIF4E on Dcp2 decapping rate under single-hit kinetics (k_obs_). When determining k_obs_, it is necessary to hold the concentration of Dcp2 at a level that is saturating (39). To determine at which point Dcp2 becomes saturating, we incubated the m7G-capped RNA oligos with increasing concentrations of Dcp2 and an excess of Xrn1 (S3 Fig). Through this experiment, we found that Dcp2 was saturating at concentrations at least 10-fold in excess of the oligo. Thus, we held Dcp2 at a 20-fold molar excess to quantitate the k_obs_. Using this molar ratio, we conducted a decapping time-course assay, wherein we incubated Dcp2 with the m7G-capped RNA oligo for various time-points before stopping the reaction. Upon the completion of the time-course, we separated the capped from the uncapped RNA species via denaturing PAGE (Fig 5A). By comparing the ratio of the capped to the uncapped species across the time-course, we found that the k_obs_ of human Dcp2 is 0.05 ± 0.01 ^min^ (Fig 5B). This rate is ~ 5X higher than the k_obs_ of the yeast Dcp1:Dcp2 heterodimer [13]. To quantify the effect of eIF4E on the rate of decapping by Dcp2, we first incubated the m7G-capped RNA oligo with a 20-fold molar excess of eIF4E, before adding Dcp2 and incubating the mixture across the same time-course as was used above (Fig 5B, C). From this experiment, we calculated the k_obs_ of Dcp2 on eIF4E bound RNA as being 0.03 ± 0.005 ^min^. Thus, while we found that eIF4E mildly impedes Dcp2 catalysis (see Fig 5A at 16 min vs 5C at 16 min), it is clear that eIF4E does not block de-capping.

## Discussion

Our results provide an additional level of mechanistic detail on the activity of Dcp2 in humans. We confirmed that this enzyme specifically removes the m7G cap from RNA in the absence of any decapping coactivators, with a turnover rate of ~20 minutes under single-order kinetics (Fig 5A). Several recent studies have reported that human Dcp2 decaps RNA in the absence of any decapping coactivators in vitro, although the extent of this activity is conflicting across various reports [24,30]. These distinctions can likely be attributed to differences in the employed experimental methodology. How additional human decapping coactivators impact the rate of decapping remains to be determined.

Surprisingly, we found that Dcp2 has an affinity for m7G-capped RNA (Figs 2A, B, 4D) that is in-line with the affinity of eIF4E for m7G-capped RNA [14,15]. In addition, we found that Dcp2 has an approximately equal affinity for 5’ triphosphate RNA (Figs 2C, 4D). These latter results are consistent with previous reports, which have found that human Dcp2 binds to both capped and 5’ triphosphate RNA, and that 5’ triphosphate RNA competes with capped RNA for Dcp2 binding [29,31]. Intriguingly, we found that Dcp2 has a slightly stronger affinity for 5’ monophosphate RNA (Figs 2D, 4D). Structural studies of yeast Dcp2 have found that this enzyme contains a bipartite active site and forms distinct ‘on’ and ‘off’ states, with only the ‘on’ state capable of catalyzing decapping [21,22,39]. How these different structures interact with these binding options will require further examination. Moreover, our results suggest that Dcp2 binds to RNA cooperatively and/or nonspecifically in all of the tested conditions (see Hill coefficients in Figs 2, 4), which is in-line with previous findings obtained using yeast Dcp2 [38]. In addition, our results imply that Dcp2 requires a functional NUDIX domain to bind RNA (Fig 3), which has also been suggested by others [38,40]. It should be noted that Mg^2+^ is widely understood to influence protein-RNA interactions. While we found that Mg^2+^ does not majorly influence the binding of Dcp2 (Fig 2C, E), nor eIF4E (Fig 4A, S3B Fig) to RNA, these experiments cannot exclude the possibility that Mg^2+^ will influence how Dcp2 and eIF4E interact on an RNA scaffold.

Perhaps most surprising, we found that bound eIF4E increases the affinity of Dcp2 for the RNA by ~2-fold (Fig 4C). It is currently unknown how Dcp2 is recruited to the mRNA 5’ ends. While our data is limited to in vitro examination, our results indicate that eIF4E may play a role in this process. This makes intuitive sense, as eIF4E serves as the 5’ end beacon for the translation initiation apparatus. Our results suggest that Dcp2 binding results in eIF4E dissociation (Fig 4A). This model is consistent with both our finding that pre-bound eIF4E does not significantly impact the rate of Dcp2 decapping (Fig 5), as well as the numerous studies, which suggest that Dcp2 cannot access the cap when eIF4E is bound [10,21–23,41–45]. In conclusion, while our results bring into question the validity of the prevailing model, how exactly these two factors interact with each other on the same RNA remains to be determined. In future studies, we will further probe the mechanism of this interaction through in vivo approaches.

## Materials and methods

### Synthesis of RNA oligonucleotides

The RNA oligos used in this study were obtained from TriLink Biotechnologies. TriLink constructed these oligos via *in vitro* transcription using the CleanCap system. Full-length RNA oligos were then purified using HPLC. However, this HPLC procedure was not capable of completely purifying these oligos. In each case, the final oligo preparation contained minor amounts of contaminating RNA species. These contaminating RNAs were not included in any quantitation. To ensure that the RNA oligos only contained a single Cy5 label when appropriate, the template sequence only contained a single cysteine nucleoside at the 3’-most position. 5’ monophosphate RNA oligos were obtained by incubating the 5’ triphosphate RNA oligos with the bacterial RNA 5’ pyrophosphohydrolase, RppH (NEB cat # M0356S), then purified using silica-membrane spin columns. Each of the RNA oligos was of the sequence 5’-AGGAAAUAAGAGAGAAAAGAAGAGUAAGAAGAAAUAUAAGAGC-3’.

### Recombinant protein cloning, overexpression, and purification

Both the Dcp2 and eIF4E coding sequences was codon-optimized for bacterial expression using the GenScript codon optimization tool. Briefly, a GBlock DNA fragment was purchased, which contained an NheI cleavage site, followed by a start codon, then a 6-histidine-protein G tag, then a TEV protease cleavage site, then the optimized Dcp2 or eIF4E sequence, and ended with a XhoI cleavage site. This DNA fragment was PCR amplified using Vent Polymerase and then cloned into the pET-28c bacterial overexpression vector between the NheI and XhoI sites. Upon confirmation of correct insertions, the Dcp2 and eIF4E overexpression vectors were transformed into BL21 (DE3) cells.

Both Dcp2 and eIF4E were purified as described previously [29,41]. To purify Dcp2 or eIF4E, the BL21 strain expressing the pET28c vector containing the appropriate sequence was streaked out onto LB plates containing 30 ug/mL Kanamycin. The next day, a single colony was picked and used to inoculate 5 mL of LB liquid media supplemented with 30 ug/mL of Kanamycin. This culture was grown overnight shaking at 37°C. The next morning, 2.5 mL of the overnight growth was added to 250 mL of pre-warmed TB media supplemented with 30 ug/mL of Kanamycin. This flask was grown shaking at 37°C until the O.D. had reach 0.4–0.6. At this point, the flask was taken out of the incubator and left standing at room-temperature for approximately 10 minutes, at which point IPTG was added to 1 mM for Dcp2, and 0.5 mM for eIF4E. The flask was then shaken at 19°C for ~18–24 hours. After this time, the growth was spun down for 20 minutes at 4000 rpm at 4°C. The media was poured off and the wet bacterial pellet was stored at −80°C for at least 20 minutes. First, the pellet was resuspended in either Dcp2 Lysis Buffer (500 mM NaCl, 40 mM Tris pH 7.9, 2 mM β-ME, 5% Glycerol, 25 mM Imidazole, 1% Triton X-100, 0.5 M Urea, 0.5 mg/mL Lysozyme, 1 pill of Roche EDTA Free protease inhibitor cocktail per 50 mL Lysis Buffer), or eIF4E Lysis Buffer (200 mM KCl, 20 mM HEPES pH 7.5, 1 mM DTT, 10% Glycerol, 25 mM Imidazole, 0.5 mg/mL Lysozyme, 1 pill of Roche EDTA Free protease inhibitor cocktail per 50 mL Lysis Buffer). The slurry was kept on ice for 30 minutes. During this time, the French-press was prepped. After the 30 minutes had passed, the cell slurry was passed through the ice-cold French-press 3 times at ~1500 psi. The crude lysate was then spun down for 20 minutes at 30,000g. The clarified lysate was quickly removed from the pellet and added to Ni-NTA resin that had been pre-equilibrated with the appropriate Lysis Buffer. The bead slurry was mixed gently at 4°C for 1 hour, then added to an empty poly-prep chromatography gravity-flow column. Once all of the beads had settled, the beads were washed with 10 column volumes of the Dcp2 Lysis Buffer, followed by 10 column volumes of either Dcp2 wash buffer (200 mM NaCl, 40 mM Tris pH 7.9, 2 mM β-ME, 5% Glycerol, 25 mM Imidazole), or 20 volumes of eIF4E Lysis Buffer. The proteins were then eluted using either Dcp2 elution buffer (Wash Buffer with 250 mM Imidazole), or eIF4E elution buffer (Lysis Buffer with 250 mM Imidazole). One mL elution fractions were collected and Bradford reagent was used to gauge where protein elution started and stopped. The eluent was collected and then, dialyzed overnight at 4°C into the appropriate elution buffer supplemented with ~60 units TEV protease. In the morning, the eluate was added to Ni-NTA resin that had been pre-equilibrated with either Dcp2 Wash Buffer or eIF4E Lysis Buffer and the slurry was mixed gently at 4°C for 1 hour, then added to an empty poly-prep chromatography gravity-flow column. The flow-through was collected and then concentrated to 0.5 mL using a Pierce PES protein concentrator and then the buffer was switched to a low-salt cation exchange buffer (50 mM KCl, 20 mM HEPES pH 7.3, 10% Glycerol, 1 mM DTT, and 0.05% Tween-20 for Dcp2) using a PD10 column. The protein was then captured via FPLC using a HiTrap SP HP cation exchange column and eluted using a gradient of cation exchange elution buffer (1 M KCl, 20 mM HEPES pH 7.3, 10% Glycerol, 1 mM DTT, and 0.05% Tween-20 for Dcp2). The fractions corresponding to the largest elution peak were collected and concentrated to 0.5 mL using a Pierce PES protein concentrator and the buffer was switched to storage buffer (200 mM KCl, 20 mM HEPES pH 7.3, 10% Glycerol, 1 mM DTT, and 0.05% Tween-20 for Dcp2). The concentration of the purified protein was measured using a DC assay and the purity was gauged via SDS-PAGE with the protein bands being visualized using a Coomassie stain. The protein preparation was then aliquoted for single-use, flash frozen using LN2 and stored at −80°C.

Mutant Dcp2 was purified using an identical protocol as wild-type Dcp2.

### RNA decapping assays and first-order rate constant measurement

To measure RNA decapping, 50 nM of each RNA oligo was incubated with 1 μM of purified wild-type or mutant Dcp2, and 1 μM of purified eIF4E when specified in a decapping reaction buffer (100 mM NaCl, 50 mM Tris-Cl pH 7.9, 10 mM MgCl_2_ or MnCl_2_, 1 mM DTT, 100 µg/ml BSA) at 37°C. This concentration of eIF4E was selected, as at this concentration, ~ 93% of all capped oligos are bound to eIF4E [46]. In all cases where Xrn1 was used, 0.5 units of yeast Xrn1 (NEB cat # M0338L) was added to the reaction. To stop the reaction, the mixture was added to 2X RNA stop solution/loading dye (40 mM Tris-base, 20 mM Na2EDTA, 0.2% sodium dodecyl sulfate, 0.05% bromophenol blue and 0.05% xylene cyanol in formamide). To resolve the capped from the uncapped RNA species, the mixture was loaded onto either a 15% polyacrylamide 8M Urea sequencing gel and run for 16 hours at 300V, or an 8% denaturing PAGE gel and run for 30 minutes at 200V. The products were resolved using an Amersham Typhoon Phophor imager and digitally captured using the associated Amersham Typhoon software. Decapping efficiencies were calculated using ImageJ by comparing the signal intensity of the capped RNA species to the signal intensity of the uncapped or decayed RNA species. In Fig 1C, the decapping efficiency was calculated by comparing the signal intensity of the full-length RNA in the + Dcp2 conditions to the intensity of the full-length RNA in the -Dcp2 condition.

The first-order rate constant of Dcp2 (k_obs_) was measured as described previously [38]. Briefly, the decapping efficiency (% decapped) was quantitated at each time point. This data was fitted to the following model [% decapped] = C*(1-e^-k*t^), where C is the maximum % decapped value.

### MicroScale thermophoresis assay and affinity measurement

To obtain the binding strength between Dcp2 and RNA, the following protocol was conducted. First, 150 uL of an RNA binding solution was created (4 nM 3’ Cy5 labeled RNA, 100 mM NaCl, 50 mM Tris-Cl pH 7.9, 1 mM DTT, 100 µg/ml BSA, 10 mM MgCl_2_ when specified, and 80nM eIF4E when specified). Next, 20 uL of Dcp2 binding solution (6 μM of Dcp2, 100 mM NaCl, 50 mM Tris-Cl pH 7.9, 1 mM DTT, 100 µg/ml BSA) was added to the 1^st^ well. 10 uL of binding buffer was then added to wells 2–12 (100 mM NaCl, 50 mM Tris-Cl pH 7.9, 1 mM DTT, 100 µg/ml BSA, and 10 mM MgCl_2_ when specified). The Dcp2 solution was then serially diluted in the following 11 wells. These samples were loaded into premium glass capillary tubes and the set of tubes was fed into a NanoTemper Monolith X that had been set to 37°C. The samples were allowed to incubate in the 37°C Monolith for 5 minutes before the Fnorms were quantitated. The Fnorm was plotted vs. the concentration of Dcp2 in each capillary tube and the resultant EC_50_ values and Hill coefficients were then modeled by the Monolith control software [32]. When present, eIF4E was included in the RNA binding solution at 80 nM and the mixture was incubated for 30 minutes at 37°C prior to addition of Dcp2.

To calculate Fnorm, the fluorescence after thermophoresis (F_1_) was divided by the fluorescence prior to thermophoresis (F_0_). More specifically, F_1_ is the fluorescence during the heated state, while F_0_ is the fluorescence during the cold state, before the IR laser is turned on. All plots were generated using F_1_ values obtained 1.5s after the IR laser was turned on. All EC_50_ values and Hill coefficients were modeled based on the F_1_ value which displayed the greatest signal to noise ratio. There were no outliers included in the statistical analysis.

### Gel-shift assays

The gel-shift assays were conducted as described previously [45], with several modifications. For the combinatorial gel shift assays, 25 nM of oligo was incubated with either no factor, 625 nM eIF4E for 30 minutes at 24°C, or 625 nM Dcp2 for 5 minutes at 24°C, or first with 625 nM eIF4E for 30 minutes at 24°C, at which point 625 nM of Dcp2 was added and the mixture was incubated for an additional 5 minutes at 24°C. For the Dcp2 titration gel shift assays, 25 nM of oligo was incubated with a 2-fold serial dilution of Dcp2 starting at 5 μM and ending at 78 nM. All of these binding events were held in -Mg^2+^ binding buffer (100 mM NaCl, 50 mM Tris-Cl pH 7.9, 1 mM DTT, 100 µg/ml BSA, 5% Glycerol and 2 mM of m7GpppA cap analog, when specified). The various RNA species were resolved on a 6% non-denaturing PAGE gel buffered with TBE. The gels were first run for 5 minutes at 75V and 24°C, after which they were run for 20 minutes at 150V at 5°C. To visualize the bands, the gel were stained with 2X CYBER-Gold for 15 minutes at room temperature before imaging using a BioRad Gel Doc Imaging system. The relative intensity of the fraction bound singly to eIF4E was quantified using ImageJ.

For the eIF4E gel shift seen in Fig S2B, 50 nM of each oligo was incubated with either no factor or 2.5 µM of eIF4E for 30 minutes at 37°C in eIF4E binding buffer (50 mM KOAc, 20 mM Tris-OAc pH 7.9, 10 mM MgOAc, 100 µg/ml BSA) before gel-shift loading buffer was added to 1X (20 mM Tris-base, 0.05% bromophenol blue, 5% Glycerol). This mixture was resolved using an 6% polyacrylamide gel that was buffered with TBE. The products were resolved using an Amersham Typhoon Phophor imager and digitally captured using the associated Amersham Typhoon software.

## Supporting information

S1 Fig Purification of Dcp2 and oligos used in these studies. (PDF)

S2 Fig Purification and validation of human eIF4E. (PDF)

S3 Fig Dcp2 is saturating when held at a 10-fold molar excess. (PDF)

S4 Appendix DNA sequences used in study. (DOCX)
