# Supplementary figures and images for: Biochemical analysis of human eIF4E-DCP2 interaction: Implications for the relationship between translation initiation and decapping
Source: PLoS One. 2025 Aug 1;20(8):e0322271. doi: 10.1371/journal.pone.0322271 (PMC12316266; doi:10.1371/journal.pone.0322271)

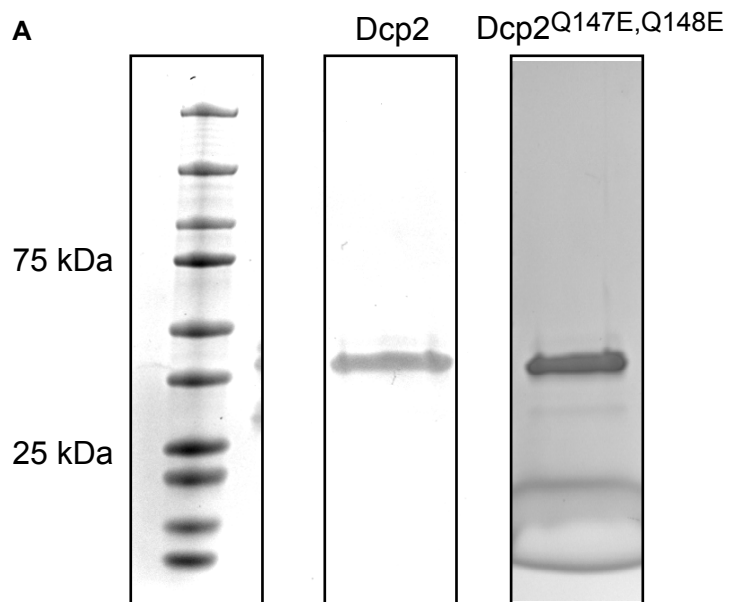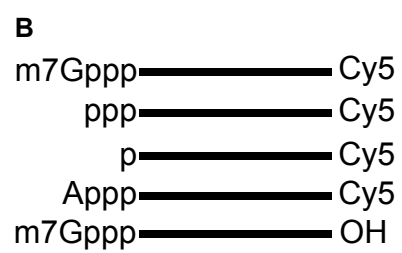

**A**

75 kDa

25 kDa

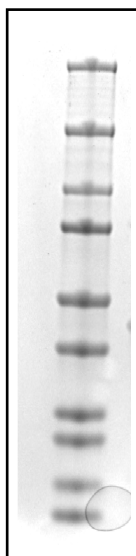

eIF4E

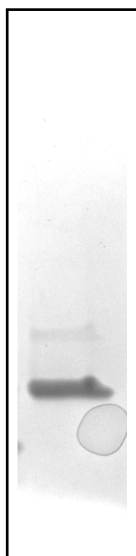**B**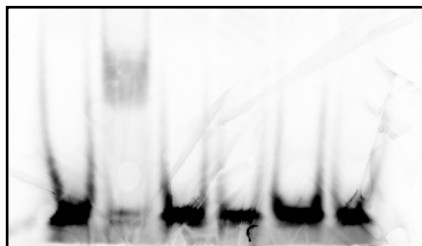

|         |        |   |     |   |      |   |
|---------|--------|---|-----|---|------|---|
| 5' end: | m7Gppp |   | ppp |   | Appp |   |
| eIF4E:  | -      | + | -   | + | -    | + |

Supplement: S1 Fig — (PDF) [file pone.0322271.s001.pdf]

**A**

75 kDa

25 kDa

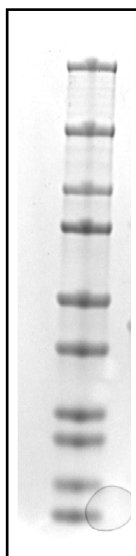

eIF4E

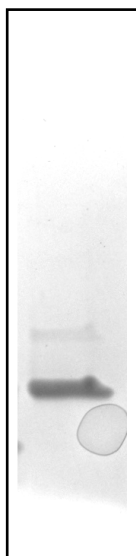**B**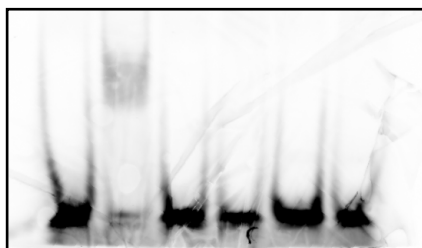

— b

— u

5' end: m7Gppp   ppp   Appp  
eIF4E:   -   +   -   +   -   +

Supplement: S2 Fig — (PDF) [file pone.0322271.s002.pdf]

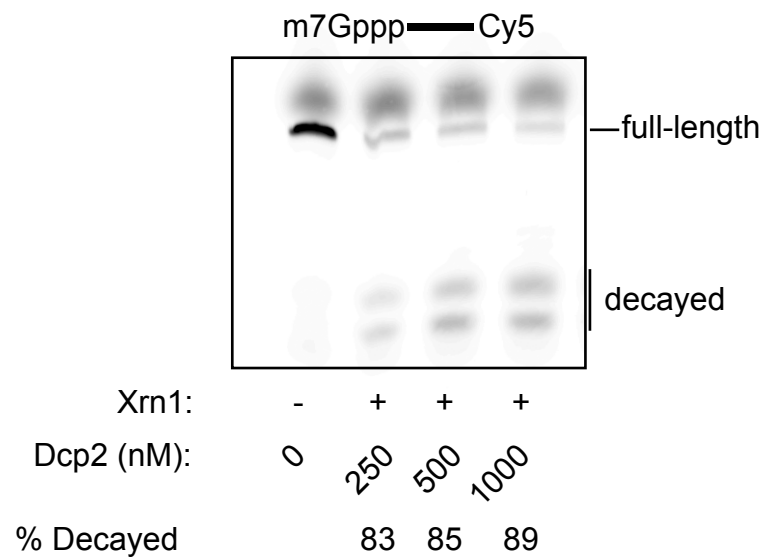

Supplement: S3 Fig — (PDF) [file pone.0322271.s003.pdf]
