# Supplementary material for: Biochemical analysis of human eIF4E-DCP2 interaction: Implications for the relationship between translation initiation and decapping
Source: PLoS One. 2025 Aug 1;20(8):e0322271. doi: 10.1371/journal.pone.0322271 (PMC12316266; doi:10.1371/journal.pone.0322271)
Supplement: S4 Appendix — (DOCX) [file pone.0322271.s004.docx]

**SUPPORTING INFORMATION FOR** ‘Translational initiation factor eIF4E does not inhibit decapping enzyme binding nor activity in vitro.’

**AUTHORS**

Zachary F. Mandell^1^ and Jeff Coller^1,2^*

^1^Department of Molecular Biology and Genetics, Johns Hopkins University, Baltimore, Maryland, 21205, USA

^2^Institute for NanoBioTechnology, Johns Hopkins University, Baltimore, Maryland 21218, USA

* To whom correspondence should be addressed. Email: [jmcoller@jhmi.edu](mailto:jmcoller@jhmi.edu).

**Appendix S4:** Show below are the DNA sequences used express the human decapping enzyme DCP2 or human translational initiation factor, eIF4E. Bolded are the ATG start codon for the purification tag and the natural ATG for each gene (downstream). The purification tag consists of 6-histidine-protein G tag, then a TEV protease cleavage site. This is then followed by a codon optimized Dcp2 or eIF4E sequence.

Dcp2 insert:

**ATG**GGTAGCTCACATCATCATCATCATCACTCTTCTGGTCTGGTCCCGCGTGGCTCGCACATGCAATACAAACTGATTCTGAACGGTAAAACGCTGAAAGGTGAAACCACGACCGAAGCAGTGGATGCGGCCACCGCTGAAAAAGTTTTCAAACAGTACGCCAACGATAATGGCGTGGATGGTGAATGGACCTATGATGACGCAACGAAAACCTACACGGTGACCGAAGGTTCCGGCGGTGAAAATCTGTACTTCCAAGGCCAT**ATG**GAGACAAAAAGGGTAGAAATACCCGGATCCGTCTTAGACGACCTGTGCTCCCGTTTCATCCTGCACATCCCGAGCGAAGAACGCGACAACGCTATCCGTGTTTGTTTTCAGATCGAGCTCGCGCATTGGTTTTACCTTGACTTCTACATGCAGAACACCCCAGGCCTGCCGCAGTGCGGCATCCGCGACTTCGCCAAGGCGGTGTTCAGCCATTGCCCGTTCCTGTTGCCGCAAGGTGAAGATGTAGAGAAGGTGCTCGACGAATGGAAAGAATACAAAATGGGTGTTCCGACCTATGGTGCGATTATCCTGGACGAGACGCTGGAAAATGTCTTGTTGGTGCAGGGTTATCTGGCGAAGAGCGGTTGGGGCTTTCCGAAGGGCAAGGTGAATAAGGAAGAGGCTCCGCATGATTGCGCGGCCCGTGAAGTGTTCGAGGAGACGGGCTTTGACATCAAAGATTACATCTGCAAAGACGACTACATTGAGTTACGTATTAATGATCAACTGGCGAGACTGTATATCATCCCGGGTATTCCGAAAGATACCAAATTCAACCCGAAGACCCGTCGTGAAATTCGCAACATTGAGTGGTTCTCCATTGAGAAGCTGCCGTGTCACCGTAACGACATGACCCCGAAGTCGAAACTGGGCCTGGCGCCAAATAAATTCTTTATGGCAATTCCATTTATCCGTCCGCTGCGCGACTGGCTGTCTCGTCGTTTCGGTGATTCCTCTGATTCCGACAACGGCTTCAGCAGCACTGGTTCGACCCCGGCGAAACCGACCGTTGAAAAACTGAGCCGCACCAAGTTCCGCCATTCTCAGCAACTGTTTCCGGATGGTAGCCCGGGCGATCAATGGGTTAAACACCGTCAGCCGCTGCAACAAAAACCGTACAACAACCATAGCGAGATGAGCGATCTGCTGAAAGGTAAAAACCAGAGCATGCGTGGTAATGGCCGTAAACAGTATCAGGATTCTCCGAACCAGAAGAAACGCACCAATGGTCTGCAACCGGCTAAGCAGCAAAACAGCCTGATGAAGTGCGAAAAAAAGTTGCACCCTCGTAAGCTGCAGGACAATTTTGAGACGGATGCAGTTTATGATTTGCCAAGCAGTTCCGAGGATCAATTATTGGAGCACGCGGAAGGTCAGCCGGTTGCGTGTAATGGCCACTGCAAGTTCCCGTTCTCATCGCGCGCATTTCTGAGCTTTAAATTTGACCACAACGCCATCATGAAGATCTTGGACCTT**TAA**

eIF4E insert:

**ATG**GGTAGCTCACATCATCATCATCATCACTCTTCTGGTCTGGTCCCGCGTGGCTCGCACATGCAATACAAACTGATTCTGAACGGTAAAACGCTGAAAGGTGAAACCACGACCGAAGCAGTGGATGCGGCCACCGCTGAAAAAGTTTTCAAACAGTACGCCAACGATAATGGCGTGGATGGTGAATGGACCTATGATGACGCAACGAAAACCTACACGGTGACCGAAGGTTCCGGCGGTGAAAATCTGTACTTCCAAGGCCAT**ATG**GCCACCGTTGAACCGGAAACGACCCCGACGACCAACCCGCCGCCGGCTGAAGAAGAAAAAACCGAAAGCAACCAGGAAGTCGCGAATCCGGAACATTATATTAAACACCCGCTGCAAAACCGTTGGGCTCTGTGGTTTTTCAAAAACGATAAATCAAAAACGTGGCAGGCGAACCTGCGCCTGATTTCGAAATTTGATACCGTGGAAGACTTCTGGGCACTGTATAACCACATCCAACTGAGCTCTAATCTGATGCCGGGTTGCGATTACAGCCTGTTTAAAGACGGCATTGAACCGATGTGGGAAGATGAGAAAAACAAACGTGGCGGTCGCTGGCTGATCACGCTGAACAAACAGCAACGTCGCTCTGATCTGGACCGTTTTTGGCTGGAAACCCTGCTGTGCCTGATTGGCGAAAGTTTCGATGACTACTCCGATGACGTTTGTGGTGCGGTGGTTAATGTCCGTGCCAAAGGCGATAAAATTGCAATCTGGACGACCGAATGTGAAAACCGCGACGCCGTCACCCATATCGGCCGTGTGTATAAAGAACGCCTGGGTCTGCCGCCGAAAATTGTTATCGGCTACCAGAGCCACGCAGATACGGCGACCAAATCGGGCAGCACCACCAAAAATCGTTTCGTTGTG**TGA**
